# Predicting Long-Term Prognoses and Grading Platinum Sensitivity Using a Novel Progression-Free Interval Criterion in Ovarian Clear Cell Carcinoma: A Multi-Institutional Cohort Study

**DOI:** 10.3390/cancers14071746

**Published:** 2022-03-29

**Authors:** Cheng-Yang Chou, Wen-Fang Cheng, Min-Yu Chen, Hao Lin, Chih-Ming Ho, Yao-Ching Hung, Lee-Wen Huang, Po-Hui Wang, Mu-Hsien Yu, Yu-Fang Huang

**Affiliations:** 1 Department of Obstetrics and Gynecology, National Cheng Kung University Hospital, College of Medicine, National Cheng Kung University, Tainan 704, Taiwan; chougyn@mail.ncku.edu.tw; 2 Department of Obstetrics and Gynecology, National Taiwan University Hospital, Taipei 100226, Taiwan; wenfangcheng@yahoo.com; 3 Department of Obstetrics and Gynecology, Chang Gung Memorial Hospital, College of Medicine, Chang Gung University, Taoyuan 333, Taiwan; e12013@cgmh.org.tw; 4 Department of Obstetrics and Gynecology, Kaohsiung Chang Gung Memorial Hospital, Chang Gung University College of Medicine, Kaohsiung 83301, Taiwan; haolin@cgmh.org.tw; 5 Gynecologic Cancer Center, Department of Obstetrics and Gynecology, Cathay General Hospital, Taipei 106, Taiwan; cmho@cgh.org.tw; 6 School of Medicine, Fu Jen Catholic University, New Taipei 242, Taiwan; 7 Department of Obstetrics and Gynecology, China Medical University Hospital, China Medical University, Taichung 404, Taiwan; d6375@mail.cmuh.org.tw; 8 Department of Obstetrics and Gynecology, Shin Kong Wu Ho-Su Memorial Hospital, Taipei 111, Taiwan; m002057@ms.skh.org.tw; 9 Department of Obstetrics and Gynecology, Chung Shan Medical University Hospital, Institute of Medicine, Chung Shan Medical University, Taichung 40201, Taiwan; wang0860@csmu.edu.tw; 10 Department of Obstetrics and Gynecology, Tri-Service General Hospital, National Defense Medical Center, Taipei 114, Taiwan; hsienhui@ms15.hinet.net

**Keywords:** ovarian cancer, clear cell carcinoma, prognostic factors, platinum sensitivity, post-progression therapy

## Abstract

**Simple Summary:**

Ovarian clear-cell carcinoma is a unique subtype of epithelial ovarian cancer. This collaborative study aimed to provide important information regarding patient demographics and treatment-associated prognostic factors in ovarian clear-cell carcinoma in Asia. Given the difference in prognoses between early- and advanced-stage cohorts, varying predictors between these two cohorts were clarified in separate analyses. Efforts should be made to promote early diagnosis and the inclusion of advanced-stage patients in clinical trials. Additionally, early recognition of poor responders to platinum-based chemotherapy and those with cancer progression occurring within seven months after completing primary chemotherapy should be emphasized in research and clinical settings. Our findings suggest that treatment selection for patients with platinum-resistant/refractory features (e.g., novel biomarkers) or relapsed disease should be based on the results of ongoing clinical trials for ovarian clear-cell carcinoma. Our results (if confirmed) will guide and inform treatment recommendations for patients at risk for poor prognosis.

**Abstract:**

This large-scale study aimed to determine the long-term influences of potential prognostic predictors and progression-free interval (PFI) criteria for grading platinum-sensitivity in ovarian clear cell carcinoma (OCCC). We retrospectively reviewed the medical records of OCCC patients presenting at nine tertiary centres (1995–2015), and evaluated patient characteristics, therapeutic factors, clinical outcomes, and hazard ratios for disease progression and death. We enrolled 536 patients (median follow-up, 36.6 months) and developed newly defined distributions of PFIs (seven and 14 months) for grading platinum sensitivity. In the multivariate model, preoperative CA125 levels and chemo-response independently predicted early-stage progression-free survival (PFS) risk. Post-progression cytoreduction correlated with reduced mortality risk. No unfavourable outcomes were observed with respect to coexisting endometriosis, fertility-sparing strategies, or platinum-based regimens. A PFI of <7 months, the strongest predictor of both post-progression mortality and second relapse risks, correlated with chemo-resistance, advanced tumour stage, and shortened post-progression survival. Chemotherapy regimens commonly used in front-line or relapse settings were limited in improving prognoses, especially in the advanced-stage cohort. Clinical trials of novel targeted agents and/or innovative biomarkers for chemoresistance should be comprehensively investigated and offered early to advanced-stage patients or those with OCCC progression occurring within seven months after receiving chemotherapy.

## 1. Introduction

Epithelial ovarian cancer (EOC) is the leading cause of death among patients with gynaecological cancers [1,2,3]. EOC comprises the following four major histological subtypes: high-grade serous, endometrioid, clear cell, and mucinous carcinomas. Ovarian clear cell carcinoma (OCCC) is clinically, cellularly, and molecularly unique [4,5,6]. Somatic AT-rich interaction domain 1A (*ARID1A*) mutations are present in up to 55% of OCCC tumours, and concomitant activation of the phosphoinositide 3-kinase (PI3K) catalytic subunit promotes tumour growth [7,8]. In addition to exhibiting distinctive features when compared with the most commonly occurring high-grade serous subtype, the histological distribution of OCCC in Asia differs from that in Western countries. More specifically, OCCC comprises 13–28% of EOC cases in Asia [5,9,10,11,12], which is considerably higher than the 5–10% incidence noted in Western countries. The reasons for this difference are speculative and unknown. OCCC could be related to endometriosis, as articulated in a controversial theory regarding the protective role of endometriosis in OCCC prognoses [13,14,15]. OCCC is more commonly detected at early stages; in the advanced stage, OCCC is considered a high-grade malignancy with chemo-resistant features [16,17,18].

EOC is treated with cytoreductive surgery followed by cytotoxic chemotherapy. In recent decades, platinum–paclitaxel has been established as the global standard chemotherapy backbone in the development of novel treatments for EOC [19,20,21]. Postoperative chemotherapy is recommended even for early-stage OCCC patients, with the same regimen as that for other EOC subtypes [19]. Taxanes, which are not covered under the Taiwan national health insurance scheme for early-stage EOC, may be unaffordable for many patients. Thus, the clinical outcomes of early-stage OCCC patients treated with chemotherapeutic combinations other than platinum–paclitaxel doublets may differ from those of platinum–paclitaxel users, based on findings reported in the current literature.

For relapsed EOC, grading platinum sensitivity by the distribution of 6- and 12-month progression-free intervals (PFI) has been adopted in clinical guidelines, and is relevant to the selection of second-line regimens [20,21,22]. Owing to the chemo-resistant features of OCCC, individualized PFI criteria (i.e., PFI extended to 12 and 18 months) have been proposed [21]. However, no definitive criteria have been established considering the rarity of OCCC and the sparsity of the literature to date.

These unresolved issues in the management of OCCC remain to be addressed. Hence, this multi-institutional study primarily aimed to determine the predictive roles of previously defined PFIs, coexisting endometriosis, and primary or post-progression treatments on survival, and to identify the surgical prognostic factors in early-stage OCCC. Our secondary objective was to demonstrate the correlation between clinicopathological factors and platinum sensitivity according to different PFI criteria.

## 2. Materials and Methods

### 2.1. Participants

This retrospective large-sample study included OCCC patients who underwent primary surgery at nine tertiary medical centres in Taiwan between 1 January 1995 and 31 December 2015. These nine centres provide cytoreductive surgery service by gynaecologic oncologists with surgical expertise for maximal tumour resection. Patients aged 20–90 years with histologically confirmed OCCC were considered eligible. Front-line platinum-based chemotherapies with various non-platinum agents as preoperative and/or postoperative therapy were recorded. Pregnant patients, those with a synchronous non-gynaecologic malignancy at diagnosis, those with non-OCCC histology, and/or those undergoing front-line bevacizumab treatment were excluded.

This study adhered to the tenets of the Declaration of Helsinki and the research protocol was approved by the National Cheng Kung University Hospital Institutional Review Board. The requirement for informed consent was waived owing to the retrospective nature of the study and the difficulty in re-contacting patients.

### 2.2. Data Collection and Outcome Assessments

**Figure 1:**
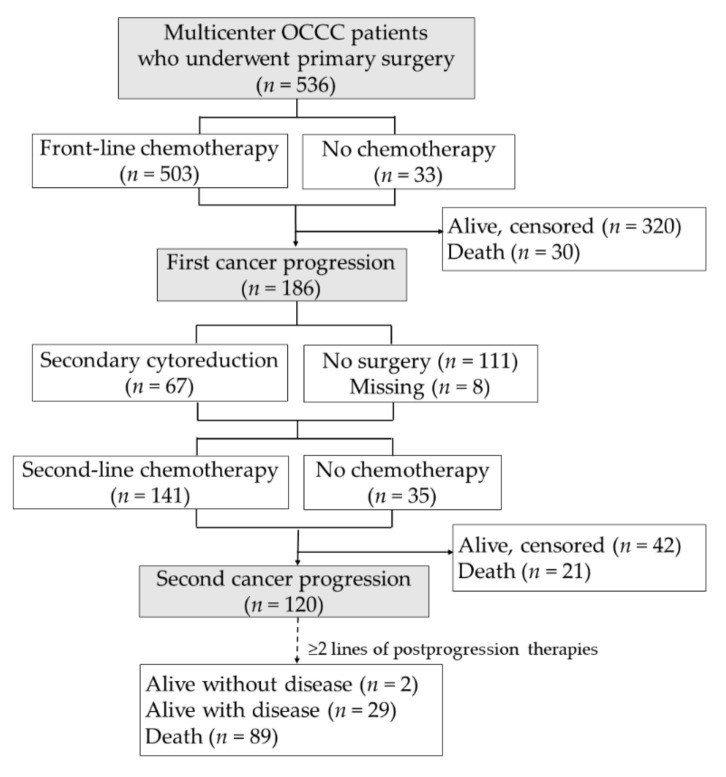
Treatment algorithm of the enrolled patients after undergoing primary treatments (*n* = 536).

The treatment algorithm for the enrolled patients is illustrated in Figure 1. Medical records were reviewed, and information on the patients’ clinical characteristics, pre-treatment CA125 levels, pathologically confirmed coexisting endometriosis, primary surgery, front-line chemotherapy, treatment outcomes, post-progression surgery, and/or salvage therapy were extracted. Residual disease (RD) (classified as tumours sized <1 and ≥1 cm) during cytoreductive surgery were categorized as ‘optimal’ and ‘suboptimal’, respectively. Follow-up records were reviewed through 31 May 2018. Overall survival (OS) was calculated according to the date of diagnosis, and progression-free survival (PFS) and PFI were determined based on the date of last contact or progression following front-line chemotherapy. Post-progression survival (PPS) was determined based on the date of the first relapse or persistence to the date of last contact or death. ‘PFS2-PFS’ was defined as the duration from the date of the first relapse or persistence to the next progression or death, whichever comes first.

### 2.3. Statistical Analyses

Continuous variables are expressed as means ± standard deviations or as medians ± interquartile ranges (or ranges, as appropriate) following normality testing, whereas categorical variables are presented as frequencies and percentages. Data were analysed using SPSS software (version 21.0, IBM Corp., Armonk, NY, USA) [23]. The receiver operating characteristic curve–determined cut-off value for cancer antigen 125 (CA125) was optimised for diagnostic sensitivity and specificity to predict cancer progression or death. Survival was estimated using the Kaplan–Meier method and was compared using the log-rank test. Statistical significance was set at two-sided *p* < 0.05. Cox proportional hazards models were used to estimate hazard ratios (HRs) and 95% confidence intervals (CIs). The grading of platinum sensitivity was assessed by comparing PFS2-PFS following second-line platinum-based therapy between patient subgroups with different PFI distributions [21]. The independent effects of the clinical prognostic factors on survival and disease progression were analysed in multivariate analyses.

## 3. Results

### 3.1. Patient Demographics

#### 3.1.1. Complete OCCC Cohort

Patient medical and demographic characteristics are described in Table 1. This study enrolled newly diagnosed patients (*n* = 536) aged 23–90 years (mean, 50.0 ± 9.9). Only 16 (3.0%) patients were older than 70 years; 158 (29.5%) were diagnosed at ≤45 years old.

The median age at menarche was 13.0 years (range, 10.0–18.0). Normal CA125 levels (≤35 U/mL) were observed in 102 (31.7%) early-stage OCCC patients and in six (4.4%) advanced-stage OCCC patients. CA125 levels were elevated at diagnosis in only 138 (44.8%) stage I OCCC patients. We noted that 30.4% of the patients had never conceived. Among those who gave birth, the mean number of deliveries was 1.5 ± 1.3 (range, 1–6). The most common symptoms of OCCC were abdominal bloating and abdominal pain. Other symptoms included urinary frequency, dysuria, and constipation.

A total of 371 (69.8%) patients were classified as having FIGO (International Federation of Gynecology and Obstetrics) stage I/II OCCC, whereas 150 (27.3%) presented at stages III/IV, and 15 (2.8%) had unknown-stage OCCC. Coexisting endometriosis was pathologically confirmed in 209 (39.0%) patients. Preoperative CA125 levels did not differ between patients with and without endometriosis, and 78.9% of the patients with endometriosis and 64.9% of the patients without endometriosis were diagnosed at an early stage (*p* = 0.001) (Table S1). Patients with endometriosis were 4.2 years younger, on average, than those without endometriosis (*p* = 0.002), and had lower gravidity and parity (difference, −0.7 and −0.6, respectively; both *p* < 0.001). Coexisting endometriosis was associated with early-stage cancer, pre-menopausal status, and RD sized <1 cm. However, endometriosis-associated OCCC was unrelated to preoperative CA125 levels, chemo-response, a PFI of seven months, and/or front-line/relapse chemotherapy regimens.

Fertility-sparing surgery was performed in 19 early-stage OCCC patients. RD sized <1 cm was achieved during primary surgery in 429 (87.6%) patients. There were 28 patients, including 19 at the early stage, who did not receive front-line chemotherapy as they presented with very early-stage disease, experienced a severe infection after primary surgery, or had poor performance status. A total of 354 (66.0%) and 120 (22.4%) patients received postoperative platinum–paclitaxel or platinum–cyclophosphamide doublet chemotherapy, respectively (Table 1). Chemotherapy combinations (5.4%) other than platinum–paclitaxel and platinum–cyclophosphamide (e.g., platinum–ifosfamide, platinum–doxorubicin, or platinum–adriamycin–cyclophosphamide) were rarely prescribed. Most patients underwent ≥6 cycles of chemotherapy. The median follow-up period was 36.6 months (range, 0.4–246). During follow-up, 376 (74.7%) patients had a PFI of ≥6 months and 127 (25.2%) had a PFI of <6 months.

#### 3.1.2. Relapse Cohort

In total, 186 (34.7%) patients had a first relapse, 120 (22.4%) had a second relapse, and 140 (26.1%) died during the study period. Of these with a first relapse, 67 (36.0%) underwent surgical resection, while 87 (46.8%) received chemotherapy other than platinum–paclitaxel/platinum–cyclophosphamide. Treatment failure and deaths (98.3%) was observed after several lines of therapies in 120 patients with a second cancer progression.

A total of 5.0 ± 3.4 and 1.2 ± 2.6 chemotherapy cycles were administered in patients with first and second relapses, respectively. The various regimens for relapsed disease included single-agent and doublet or triplet chemotherapy, such as pegylated liposomal doxorubicin, topotecan, weekly paclitaxel, platinum–pegylated liposomal doxorubicin, platinum–gemcitabine, platinum–irinotecan, ifosfamide–etoposide, platinum–paclitaxel–gemcitabine, and platinum–adriamycin–cyclophosphamide regimens.

### 3.2. Grading Platinum Sensitivity by PFI

The grading of platinum sensitivity among patient subgroups with different distributions of PFIs is shown in Table 2. A total of 85 patients were included in this analysis. The PFS2-PFS risks did not significantly differ between subgroups with PFIs of 6–12 months and <6 months after relapse therapy with a platinum-based regimen (Figure S1). Similar analyses were performed in the following comparative PFI distributions: 12 and 18, 11 and 18, 10 and 18, 9 and 18, 8 and 16, or 7 and 14 months. Only the relapsed subgroups with PFI 7–14 and ≥14 months had a significantly reduced risk of a second progression after resuming platinum-based chemotherapy (HR, 0.53; 95% CI, 0.29–0.97, and HR, 0.26; 95% CI, 0.13–0.50, respectively). Moreover, we conducted additional analyses on the following comparative PFI distributions: between 7 and 12, 7 and 16, or 7 and 18 months. The PFS2-PFS risks did not significantly differ in these secondary comparisons.

### 3.3. PFI-Associated Factors

The associations between PFIs of either 7 or 12 months and the clinicopathological factors of 503 patients treated with front-line chemotherapy are presented in Table 3. Patients with a PFI of <7 months had preoperative CA125 levels of ≥114.5 U/mL, advanced-stage disease, RD sized ≥1 cm, <6 cycles of front-line chemotherapy, and poor response to front-line chemotherapy; these patients were also more likely to receive post-progression chemotherapy regimens other than platinum–paclitaxel doublets. Patients with a PFI of <12 months more frequently presented with preoperative CA125 levels of ≥114.5 U/mL, advanced-stage disease, RD sized ≥1 cm, poor response to front-line chemotherapy, tumour resection following the first relapse, and post-progression chemotherapy regimens other than platinum–paclitaxel doublets.

No significant differences were observed upon evaluation of a PFI of seven months in relation to age, menopausal status, coexisting endometriosis, front-line chemotherapy regimens, or tumour resection following the first relapse. No significant differences were observed between a PFI of 12 months and the following factors: age, menopausal status, coexisting endometriosis, front-line chemotherapy regimens, or <6 cycles of front-line chemotherapy.

### 3.4. Stage-Associated Factors

Associations between tumour stage and patient demographics are presented in Table S2. Patients with advanced-stage OCCC more frequently presented with preoperative CA125 levels of ≥114.5 U/mL, endometriosis absence, RD sized ≥1 cm, platinum–paclitaxel doublets, poor chemo-response, a PFI of <7 months, and a waiver of surgical resection. These patients were also more likely to receive post-progression chemotherapy regimens other than platinum–paclitaxel doublets. No significant differences were noted for disease stage with respect to age, menopausal status, or cycles of chemotherapy.

### 3.5. Clinical Outcomes

#### 3.5.1. Stage-Associated and Treatment-Associated Survival

**Figure 2:**
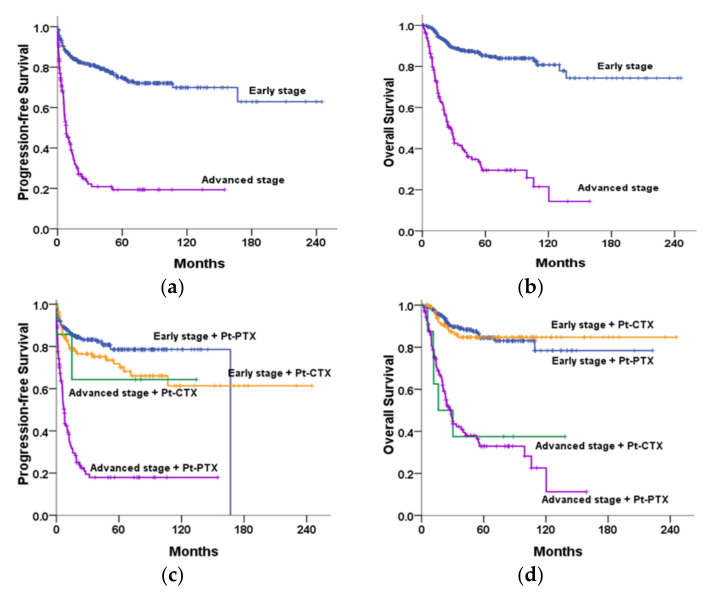
Kaplan–Meier curves. (**a**) Progression-free survival (PFS) and (**b**) overall survival (OS) significantly differed between patients with early- (*n* = 371) and advanced-stage OCCC (*n* = 150). (**c**) PFS and (**d**) OS significantly differed between the subgroups stratified by stage plus platinum-based doublets.

The survival curves for all OCCC patients stratified by stage alone or stage in combination with various chemotherapeutic regimens are illustrated in Figure 2. The median PFS (7.9 months vs. not reached, *p* < 0.001) and OS (27.1 months vs. not reached, *p* < 0.001) were significantly shorter in the advanced-stage OCCC subgroup than in the early-stage subgroup (Figure 2a,b). The five-year PFS metrics for the early- and advanced-stage subgroups were 74.9% and 19.3%, whereas the five-year OS metrics were 85.3% and 29.5%, respectively.

The median PFS and OS durations were shorter in the advanced-stage OCCC subgroup treated with platinum–paclitaxel (*n* = 124) than in the early-stage OCCC subgroup treated with platinum–paclitaxel (*n* = 223; median PFS, 16.1 months vs. not reached, *p* < 0.001; median OS, 43.7 months vs. not reached, *p* < 0.001; Figure 2c,d). No significant differences in the PFS or OS were noted between the two most commonly prescribed regimens among early-stage OCCC patients.

#### 3.5.2. Survival Curves following the First Relapse

**Figure 3:**
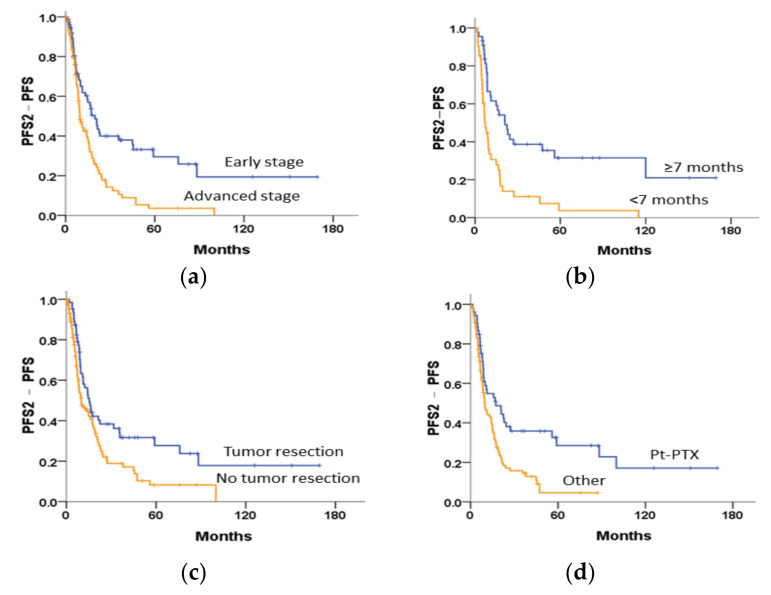
Kaplan–Meier curves. In the relapsed subgroup, PFS2-PFS was tested using the log-rank test, stratified by (**a**) stage (*p* < 0.001), (**b**) progression-free interval of 7 months (*p* < 0.001), (**c**) tumour resection (*p* = 0.009), and (**d**) chemotherapeutic regimens for the first relapse (*p* = 0.001).

The median PFS2-PFS was significantly shorter in the advanced-stage OCCC subgroup than in the early-stage OCCC subgroup (5.5 vs. 6.7 months, *p* < 0.001; Figure 3a). Similar results were found in the following subgroups: PFI < 7 months vs. PFI ≥ 7 months (8.9 months vs. 22.5 months, *p* < 0.001; Figure 3b), patients with and without tumour resection (5.3 months vs. 7.6 months, *p* = 0.009; Figure 3c), and patients treated with regimens other than platinum–paclitaxel following the first relapse (4.6 months vs. 13.7 months, *p* = 0.001; Figure 3d). The median PPS was significantly shorter in patients with a PFI of < 7 months (14.1 months vs. 45.0 months, *p* < 0.001) than in patients with a PFI ≥ 7 months (Figure S1).

### 3.6. Univariate and Multivariate Analyses

#### 3.6.1. Complete OCCC Cohort

In univariate analyses, a pre-treatment CA125 level of ≥114.5 U/mL, advanced-stage OCCC, RD sized ≥1 cm, the use of platinum–paclitaxel doublets in a front-line setting, and poor chemo-response were significantly associated with a high risk of disease progression and death (Table S3). Patients who underwent ≥six cycles of front-line chemotherapy had a lower risk of death, whereas those with a PFI of <7 months and those using post-progression chemotherapeutic regimens other than platinum–paclitaxel had a higher risk of death.

In the multivariate-adjusted model, a PFI of <7 months was the strongest predictor of death (HR, 7.85; 95% CI, 3.63–16.94; Table 4). A CA125 level of ≥114.5 U/mL (HR, 1.57; 95% CI, 1.03–2.40), advanced-stage OCCC (HR, 3.21; 95% CI, 1.98–5.22), and poor chemo-response (HR, 4.56; 95% CI, 2.88–7.23) were identified as independent predictors of the risk of cancer progression. Age was an independent predictor of cancer-associated death (HR, 2.98; 95% CI, 1.60–5.57).

#### 3.6.2. Early-Stage OCCC Cohort

Of the 19 early-stage OCCC patients who underwent fertility-sparing surgery, four (21.1%) developed disease progression and one (5.3%) died. No deterioration in terms of cancer progression and no cases of death were found in those treated with this strategy compared with those treated with radical surgery (cancer progression, 22.4%; death, 13.9%). In univariate analyses, fertility-sparing surgery (HR, 0.91; 95%CI, 0.33–2.48) and surgical spills or positive cytology (HR, 0.68; 95% CI, 0.43–1.07) did not significantly correlate with the risk of disease progression. Poor chemo-response (PFS: HR, 23.95; 95%, 13.67–41.97; OS: HR, 18.29; 95%, 9.91–33.75) and RD ≥ 1 cm (PFS: HR, 2.90; 95% CI, 1.05–8.03; OS: HR, 3.96; 95% CI, 1.42–11.08) were related to the risk of cancer progression and death. However, we did not identify any independent prognostic impacts of RD sized ≥ 1 cm (PFS: HR, 1.38; 95%, 0.39–4.92; OS: HR, 0.81; 95%, 0.09–7.81) in the multivariate-adjusted model. A pre-treatment CA125 level of ≥ 114.5 U/mL and poor chemo-response during treatment were significantly associated with an elevated risk of disease progression (Table 5). A PFI of < 7 months was the strongest predictor for an increased risk of both disease progression and death. Surgical resection of relapsed lesions was associated with a low risk of death (HR 0.23; 95% CI, 0.06–0.91). No prominent prognostic impact was observed with respect to less than six cycles of chemotherapy or various chemotherapeutic regimens.

#### 3.6.3. Relapsed OCCC Cohort

In the multivariate-adjusted model, a PFI of <7 months was an independent predictor for the next progression (HR, 2.46; 95% CI, 1.54–3.93) and post-progression death (HR, 3.86; 95% CI, 2.15–6.96; Table 6). Age ≥ 50 years (HR, 2.26; 95% CI, 1.37–3.72) and advanced-stage OCCC at initial diagnosis (HR, 1.73; 95% CI, 1.01–2.96) were significantly related to the risk of cancer-associated death.

## 4. Discussion

This large-scale retrospective study demonstrated that surgical spills, positive peritoneal cytology, and fertility-sparing surgery do not adversely affect the clinical outcomes of early-stage OCCC patients. After adjustment for potential confounders, we found that prognoses are not significantly affected by coexisting endometriosis, the extent of RD, cycles and regimens of front-line platinum-based chemotherapy, or post-progression treatments within the entire study cohort. Our findings clarify the prognostic role of both front-line and post-progression chemotherapy regimens, and indicate that the current anticancer agents used in the front-line or relapse setting have limitations in terms of improving the prognoses for OCCC patients, especially those in the advanced stage. We revealed a positive correlation between a PFI of 7 months and grades of platinum sensitivity in OCCC, compatible with the role of this regimen in predicting prognoses.

The endometriosis-associated characteristics of age at diagnosis, gravidity, and parity amongst our enrolled OCCC patients were consistent with those reported in previous studies [24,25]. Preoperative CA125 levels were elevated in almost all patients with advanced-stage OCCC but were elevated in only 68.3% of those at the early stage. Among the early-stage OCCC patients, 23.5% had normal CA125 levels; this finding is similar to the results reported by Ye et al. [24]. However, we did not observe a significant difference in the CA125 levels between patients with and without endometriosis, which was more highly powered overall and within subgroups as compared to the study by Ye et al. [24]. Previous studies have revealed that patient survival is significantly associated with endometriosis in OCCC patients [14,25]. However, we demonstrated a lack of correlation between coexisting endometriosis and disease prognoses, in agreement with previous findings [14]. Moreover, we did not observe any correlation between tumour recurrence and platinum sensitivity among those with endometriosis [24].

Partly owing to the small sample size of previous OCCC cohorts [5,26], the impact of clinicopathological factors (for example, stage [27], RD sized ≥1 cm [28], fertility-sparing surgery [29,30], front-line chemotherapy regimens [16], or post-progression therapies [22,31]) on oncologic outcomes within OCCC remains inconclusive. Initial stage and RD have been reported as prognostic predictors [24,25,27,28]. Nonetheless, we found that the influence of the cancer stage on the risk of progression persisted in the multivariate analysis, although its impact on the risk of cancer-associated mortality became insignificant upon multivariate adjustment. The extent of RD may depend upon the initial stage and surgeons’ skills. RD sized < 1 cm could be achieved in 96.8% of early-stage and 65.0% of advanced-stage patients at the centres in this study (Table S2). Due to lack of data on complete tumour resection among patients with RD sized <1 cm, the impacts of complete tumour resection on OCCC prognosis could therefore not be examined. The effects of RD on prognosis did not significantly differ after adjusting for potential confounders, possibly because of the inclusion of the newly defined PFI metric and a chemo-response variable, which were the most vital factors within our analysis.

Platinum sensitivity, defined according to a PFI of six months with respect to responses to second-line chemotherapy by retrospective studies with small sample size, has been used as a metric of interest over the past decade [20,32,33]. However, the definition of platinum sensitivity or resistance based only on a timeline for all histological EOC types has been suggested to be of limited utility [21,33,34,35]. As an independent predictive clinical biomarker for PPS and next progression, a PFI of 7 months was found to be a better criterion than PFIs of 6 [19,20,36,37,38] or 12 months [21] with respect to the grading of platinum sensitivity in OCCC. Moreover, our findings support the previously delineated multiplex classification system aiming to guide clinical treatment decisions for patients with relapsed EOC more effectively [35]. Therefore, we conclude that efforts to develop OCCC treatments, especially for patients with advanced-stage and relapsed disease, should be directed towards novel targeted therapies and/or immunotherapies (such as PI3K/protein kinase B-specific small-molecule inhibitors, mechanistic targets of rapamycin [mTOR], tyrosine kinases, or combination therapies with immune checkpoint inhibitors [ICIs] targeting programmed cell death-1 (PD-1), programmed cell death ligand-1 (PD-L1), or CTLA4) [39,40,41,42,43,44,45].

More than 50% of EOC cases are classified as an early-stage disease in the Taiwan Cancer Registry, and the increasing trend in the proportion of OCCC patients [11] has drawn our attention. More than 25% of the enrolled OCCC patients in this study were diagnosed at child-bearing age, and the preservation of reproductive function is an important concern for this young population. Fertility-sparing surgery was excluded from the recommended treatment regimen for stage I OCCC more than a decade ago [36,37]. However, considering the growing body of recent evidence [19,25,27,29,30], stage IA/IC OCCC patients may be treated conservatively according to physician judgement. In this study, the prognosis for the early-stage OCCC cohort receiving postoperative chemotherapy was favourable, as distinct from that of the advanced-stage cohort. Hence, separate analyses were conducted to further explore the prognostic predictors in each of these two cohorts. To minimize bias due to the small cohort with fertility-sparing surgery and the confounding effect of the extent of cytoreduction, we included a large number of early-stage patients and assessed the influence of surgical factors on prognosis using multivariate analysis. Our findings validated the theory that disease prognosis is not significantly affected by surgical factors. These data support favourable oncologic outcomes after the implementation of fertility-sparing strategies in early-stage OCCC based on existing institutional criteria for patient selection and postoperative treatment.

However, this study had some limitations. First, we evaluated the effect of various systemic chemotherapeutic regimens in a relatively small number of advanced-stage OCCC patients. Thus, our study was insufficiently powered for subgroup analyses. The investigators of JGOG3017, a large-scale phase III randomised controlled trial, overcame this limitation by enrolling international and multi-institutional OCCC patients across all disease stages [10]. However, the cisplatin–irinotecan doublets did not show superior anticancer activity over carboplatin–paclitaxel doublets when used as front-line chemotherapy treatments. We observed consistent results regardless of the specific non-platinum drugs added to the platinum-based chemotherapy. Colombo et al. have reported an overall response rate of 80% for a PI3K/mTORC1/2 dual inhibitor combined with carboplatin and paclitaxel among 10 OCCC patients, including seven (70%) chemo-naïve patients, and a median response duration of eight months [43]. The results of this trial reinforce our findings in an advanced OCCC cohort. As advances in various post-progression therapies have been demonstrated to result in confounding effects on OS [10,20,22,31,35], post-progression treatments should be comprehensively evaluated in future studies. In the multivariate confounder-adjusted analysis performed in this study, PPS benefits owing to tumour resection at the first relapse were noted in the early-stage OCCC cohort, regardless of the administered chemotherapeutic regimen.

Current trials evaluating the use of ICIs in ovarian cancer have not demonstrated improved survival [45,46]. Combination therapies with ICIs, chemotherapy regimens, PI3K/mTORC1/2 inhibitor [39,43], tyrosine kinase inhibitors [41,42], and/or PARP inhibitors [47] may be indicated for the treatment of advanced or relapsed OCCC. To date, there have been no positive correlations reported between treatment response and potential biomarkers related to the PI3K/mTOR or PD-1/PD-L1 pathways [44,45,46]. Hence, our stricter PFI criterion of grading platinum sensitivity for OCCC may be recommended for post-treatment surveillance.

## 5. Conclusions

If confirmed, our results can guide treatment recommendations for OCCC patients predicted to have a poor prognosis. The early detection of OCCC is mandatory for the initiation and selection of effective curative treatment options. Surveillance for the early detection of progression at seven months after receiving front-line chemotherapy is warranted to predict the risk of cancer-specific death and post-progression prognosis. Patients with PFIs of <7, 7–14, or ≥14 months may be categorised as having ‘platinum-resistant’, ‘partially platinum-sensitive’, and ‘platinum-sensitive’ OCCC, respectively. Further investigation should be conducted to validate our findings. Moreover, clinical trials evaluating novel targeted agents and the utility of potential biomarkers should be carried out to elucidate the front-line and post-progression therapies for advanced-stage OCCC patients. These curative therapies, if validated, should be offered early owing to the known limited effects of chemotherapeutic agents. Thus, our findings can guide future research directions and directly inform medical guidelines.

## Figures and Tables

**Table 1:** Patient demographics.

Characteristics	OCCC Patients (*n* = 536)
Age, years, mean ± SD	50.0 ± 9.9
Menarche age, years, median ± IQR (range)	13.0 ± 1.0 (10.0–18.0)
CA125 level at diagnosis, U/mL, median ± IQR (range)	101.5 ± 324 (6.0–16,659.0)
Nulliparity	141 (30.4)
Parity, mean ± SD	1.5 ± 1.3
Menopause	279 (52.1)
Most common symptoms, *n* (%)	
Asymptomatic	43 (8.0)
Abdominal pain	112 (20.9)
Abdominal bloating	132 (24.6)
Abnormal uterine bleeding	17 (3.2)
Other symptoms	101 (18.8)
Endometriosis present pathologically	209 (39.0)
FIGO stage, *n* (%)	
I	308 (57.5)
II	63 (12.3)
III	116 (21.1)
IV	34 (6.2)
Unknown	15 (2.8)
Primary staging/cytoreduction, *n* (%)	
RD < 1 cm	429 (80.0)
RD ≥ 1 cm	61 (11.4)
Unknown	46 (8.5)
Front-line chemotherapy, *n* (%)	
Pt-PTX	354 (66.0)
Pt-CTX	120 (22.4)
Other platinum-based regimens, *n* (%)	29 (5.4)
None	33 (6.2)
Cycles of front-line chemotherapy, median ± IQR (range)	6.0 ± 1.0 (1–11)
<6, *n* (%)	134 (26.6)
≥6, *n* (%)	369 (73.4)
PFI after primary chemotherapy, *n* (%)	
≥12 months	320 (63.6)
6–12 months	56 (11.1)
<6 months	127 (25.2)
Surgical resection after the first relapse, *n* (%)	67 (36.0)
Chemotherapy after the first relapse, *n* (%)	
None	35 (18.8)
Pt-PTX	55 (29.6)
Pt-CTX	2 (1.1)
Other	87 (46.8)

CTX, cyclophosphamide; FIGO, the International Federation of Gynecology and Obstetrics; OCCC, ovarian clear cell carcinoma; PFI, progression-free interval; Pt, platinum; PTX, paclitaxel; RD, residual disease. Missing data of each variable were excluded for analysis.

**Table 2:** Degree of platinum sensitivity in OCCC patients treated with a platinum-based regimen according to the interval from the end of first-line treatment to the first relapse (*n* = 85).

PFI after Front-Line Chemotherapy to the First Relapse (Months)	No. of Patients with Pt-Based Chemotherapy	No. of Patients with the Second Relapse	Median PFS2-PFS (Months)	HR	95% CI
<6	38	34	6.8	1.00	–
≥6	47	32	20.9	0.41	0.25–0.68
6–12	16	15	9.2	0.76	0.41–1.40
≥12	31	16	24.4	0.27	0.15–0.50
<7	41	37	6.8	1.00	–
≥7	44	28	21.0	0.37	0.22–0.61
7–12	13	12	11.2	0.68	0.35–1.31
≥12	31	16	24.4	0.27	0.14–0.49
7–14	18	16	14.9	0.53	0.29–0.97
≥14	26	12	24.4	0.26	0.13–0.50
7–16	20	18	11.2	0.57	0.32–1.01
≥16	24	10	NR	0.22	0.11–0.45
7–18	23	21	11.2	0.60	0.35–1.03
≥18	21	7	NR	0.17	0.07–0.38
<8	46	41	8.5	1.00	–
≥8	39	24	23.0	0.38	0.23–0.65
8–16	15	14	8.6	0.65	0.35–1.21
≥16	24	10	NR	0.24	0.12–0.49
<10	49	44	8.5	1.00	–
≥10	36	21	24.4	0.35	0.20–0.60
10–18	15	14	8.6	0.64	0.35–1.19
≥18	21	7	NR	0.18	0.08–0.41
<12	54	49	8.6	1.00	–
≥12	31	16	24.4	0.30	0.16–0.54
12–18	10	9	8.5	0.57	0.27–1.18
>18	21	7	NR	0.18	0.08–0.41

CI, confidence interval; HR, hazard ratio; NR, not reached; OCCC, ovarian clear cell carcinoma; PFS, progression-free survival; Pt, platinum. HRs and 95% CIs were estimated using Cox proportional hazards models. The PFS2-PFS survival curves of the patient subgroups stratified by the PFI of 7 and 14 months and the progression-free interval of 6 and 12 months are illustrated in Figure S1.

**Table 3:** Correlations between patient characteristics and PFI of 7 or 12 months among the multi-institutional OCCC patients treated with front-line chemotherapy.

Variables	PFI ≥7 Months *n* = 366	PFI <7 Months *n* = 137	*p*	PFI ≥12 Months *n* = 318	PFI <12 Months *n* = 185	*p*
Age >50 years	179 (48.9)	60 (43.8)	0.271	154 (48.4)	85 (45.9)	0.584
Menopause	192 (52.5)	68 (49.6)	0.516	162 (50.9)	98 (53.0)	0.667
CA125 level ≥114.5 U/mL	126 (34.4)	88 (64.2)	<0.001	103 (32.4)	111 (60.0)	<0.001
Endometriosis present	147 (40.1)	52 (38.0)	0.628	132 (41.5)	67 (36.2)	0.424
FIGO stage			<0.001			<0.001
I	248 (67.8)	41 (29.9)		225 (70.8)	64 (34.6)	
II	43 (11.7)	17 (12.4)		39 (12.3)	21 (11.4)	
III	46 (12.6)	64 (46.7)		31 (9.7)	79 (42.7)	
IV	18 (4.9)	13 (9.5)		12 (3.8)	19 (10.3)	
Primary cytoreduction			<0.001			<0.001
Optimal (RD <1 cm)	316 (86.3)	94 (68.6)		278 (87.4)	132 (71.3)	
Suboptimal (RD ≥1 cm)	17 (4.6)	35 (25.5)		11 (3.5)	41 (22.2)	
Front-line chemotherapy			0.192			0.121
Pt-PTX	250 (68.3)	104 (75.9)		214 (67.3)	140 (75.7)	
Pt-CTX	95 (26.0)	25 (18.2)		85 (26.7)	35 (18.9)	
Other	21 (5.7)	8 (5.8)		19 (6.0)	10 (5.4)	
Cycles of front-line chemotherapy			0.005			0.068
<6	85 (23.2)	49 (35.8)		76 (23.9)	58 (31.4)	
≥6	281 (76.8)	88 (64.2)		242 (76.1)	127 (68.6)	
Response to primary chemotherapy			<0.001			<0.001
CR/PR	335 (91.5)	68 (49.6)		298 (93.7)	105 (56.8)	
SD/PD	22 (6.0)	64 (46.7)		11 (3.5)	75 (40.5)	
Tumour resection after the first relapse			0.076			0.034
No	114 (79.2)	67 (69.1)		98 (81.0)	83 (69.2)	
Yes	30 (20.8)	30 (30.9)		23 (19.0)	37 (30.8)	
Chemotherapy after the first relapse			<0.001			<0.001
Pt-PTX	33 (54.1)	16 (21.6)		29 (70.7)	20 (21.3)	
Other	28 (45.9)	58 (78.4)		12 (29.3)	74 (78.7)	

Data are presented as mean (range) or frequency (percentage). Missing data of each variable were excluded for analysis. Differences in the continuous variables were tested using the Mann–Whitney *U* test. Correlations between categorical variables were compared using Pearson’s chi-square or Fisher’s exact tests. CR, complete response; CTX, cyclophosphamide; FIGO, the International Federation of Gynecology and Obstetrics; OCCC, ovarian clear cell carcinoma; PD, progressive disease; PFI, progression-free interval; PR, partial response; Pt, platinum; PTX, paclitaxel; RD, residual disease; SD, stable disease.

**Table 4:** Multivariate analysis of prognostic factors in all OCCC patients (*n* = 536).

Variables	HR for the First Progression (95% CI)	HR for Death (95% CI)
Age (≥50 vs. <50 years)	0.89 (0.62–1.27)	2.98 (1.60–5.57)
CA125 level at diagnosis (≥ 114.5 versus <114.5 U/mL)	1.57 (1.03–2.40)	0.71 (0.34–1.47)
Endometriosis present (yes versus no)	1.00 (0.69–1.44)	0.93 (0.52–1.64)
FIGO stage (advanced versus early)	3.21 (1.98–5.22)	1.41 (0.67–2.95)
Primary staging/cytoreduction (RD ≥ 1 cm versus <1 cm)	1.30 (0.81–2.08)	1.12 (0.59–2.34)
Front-line chemotherapy (Pt-PTX versus Pt-CTX)	0.69 (0.41–1.16)	0.71 (0.25–2.03)
Cycles of chemotherapy (≥6 versus <6)	0.88 (0.59–1.33)	1.04 (0.51–2.10)
Response to chemotherapy (SD/PD versus CR/PR)	4.56 (2.88–7.23)	1.47 (0.79–2.73)
Progression-free interval (<7 months versus ≥7 months)	-	7.85 (3.63–16.94)
Surgical resection after the first relapse (yes versus no)	-	0.60 (0.29–1.23)
Chemotherapy after the first relapse (others versus Pt-PTX)	-	1.23 (0.52–2.89)

CI, confidence interval; CR, complete response; CTX, cyclophosphamide; FIGO, the International Federation of Gynecology and Obstetrics; HR, hazard ratio; OCCC, ovarian clear cell carcinoma; PD, progressive disease; PFI, progression-free interval; PR, partial response; Pt, platinum; PTX, paclitaxel; RD, residual disease; SD, stable disease.

**Table 5:** Multivariate analysis of prognostic factors in early-stage OCCC patients (*n* = 371).

Variables	HR for the First Progression (95% CI)	HR for Death (95% CI)
Age (≥50 versus <50 years)	0.72 (0.39–1.31)	2.39 (0.67–8.50)
CA125 level at diagnosis (≥ 114.5 versus <114.5 U/mL)	2.63 (1.48–4.67)	0.61 (0.17–2.19)
Endometriosis present (yes versus no)	0.74 (0.41–1.33)	0.44 (0.14–1.35)
Primary staging/cytoreduction (RD ≥ 1 cm versus <1 cm)	1.38 (0.39–4.92)	0.81 (0.09–7.81)
Front-line chemotherapy (Pt-PTX versus Pt-CTX)	0.61 (0.34–1.09)	0.48 (0.12–1.99)
Cycles of chemotherapy (≥6 versus <6)	0.76 (0.41–1.41)	2.22 (0.57–8.61)
Response to chemotherapy (SD/PD versus CR/PR)	22.77 (11.64–44.56)	1.83 (0.44–7.57)
PFI after primary chemotherapy (<7 versus ≥7 months)	-	4.82 (1.32–17.60)
Surgical resection after the first relapse (yes versus no)	-	0.23 (0.06–0.91)
Chemotherapy after the first relapse (other versus Pt-PTX)	-	1.64 (0.41–6.62)

CI, confidence interval; CR, complete response; CTX, cyclophosphamide; FIGO, the International Federation of Gynecology and Obstetrics; HR, hazard ratio; OCCC, ovarian clear cell carcinoma; PD, progressive disease; PFI, progression-free interval; PR, partial response; Pt, platinum; PTX, paclitaxel; RD, residual disease; SD, stable disease. HRs and 95% CIs were estimated using Cox proportional hazards models.

**Table 6:** Multivariate analysis of prognostic factors in OCCC patients with first relapse (*n* = 186).

Variables	HR for the Next Progression (95% CI)	HR for Post- Progression Death (95% CI)
Age (≥50 versus <50)	1.49 (0.97–2.30)	2.26 (1.37–3.72)
FIGO stage (advanced versus early)	1.50 (0.95–2.37)	1.73 (1.01–2.96)
Response to chemotherapy (SD/PD versus CR/PR)	1.33 (0.85–2.08)	1.26 (0.76–2.10)
Progression-free interval (<7 versus ≥7 months)	2.46 (1.54–3.93)	3.86 (2.15–6.96)
Surgical resection after the first relapse (yes versus no)	0.83 (0.52–1.32)	0.83 (0.50–1.41)
Chemotherapy after the first relapse (other versus Pt-PTX)	1.04 (0.62–1.75)	0.95 (0.51–1.78)

CI, confidence interval; CR, complete response; CTX, cyclophosphamide; FIGO, the International Federation of Gynecology and Obstetrics; HR, hazard ratio; OCCC, ovarian clear cell carcinoma; PD, progressive disease; PFI, progression-free interval; PR, partial response; Pt, platinum; PTX, paclitaxel; RD, residual disease; SD, stable disease.

## Data Availability

The datasets analysed in this article are not publicly available because they contain information that could compromise the privacy of the research participants. The data presented in this study are available on request from the corresponding author.

## Supplementary Materials

The following supporting information can be downloaded at: https://www.mdpi.com/article/10.3390/cancers14071746/s1, Table S1: Clinico-pathological characteristics of multi-institutional ovarian clear cell carcinoma (OCCC) patients with and without endometriosis; Table S2: Clinico-pathological characteristics of multi-institutional ovarian clear cell carcinoma (OCCC) patients with early- and advanced-stage disease; Table S3: Univariate analysis of prognostic factors in the complete ovarian clear cell carcinoma (OCCC) cohort; and Figure S1: Kaplan–Meier curves. (a) Progression-free survival 2 (PFS2)-PFS and (b) post-progression survival.
