# Supplementary material for: Predicting Long-Term Prognoses and Grading Platinum Sensitivity Using a Novel Progression-Free Interval Criterion in Ovarian Clear Cell Carcinoma: A Multi-Institutional Cohort Study
Source: Cancers (Basel). 2022 Mar 29;14(7):1746. doi: 10.3390/cancers14071746 (PMC8997040; doi:10.3390/cancers14071746)
Supplement: Supplementary file 1 [file cancers-14-01746-s001.zip › cancers-1607180-supplementary.pdf]

Supplementary Materials

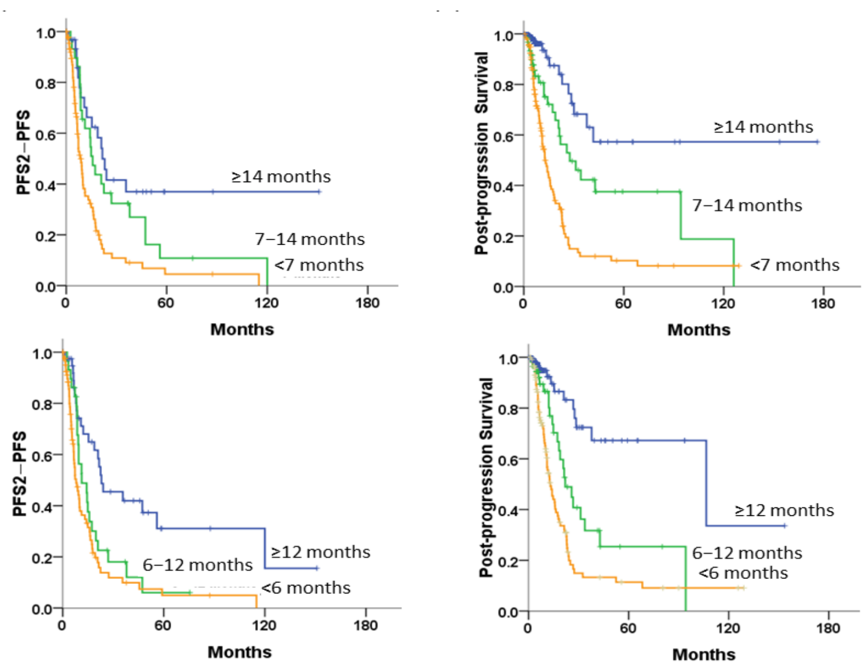

(a) (b)

Figure S1. Kaplan–Meier curves. (a) Progression-free survival 2 (PFS2)-PFS and (b) post-progression survival significantly differed between the subgroups divided by progression-free interval distributions of 7 and 14 months (both *P*<0.001, respectively). However, the PFS2-PFS did not show a significant disparity between a progression-free interval of <6 months and 6–12 months (left lower panel).

Table S1. Clinico-pathological characteristics of multi-institutional ovarian clear cell carcinoma (OCCC) patients with and without endometriosis.

Variables	Endometriosis presence n = 209	Endometriosis absence n = 268	P
Age >50 years	84 (40.4)	146 (54.9)	0.002
Menopause	94 (45.2)	150 (56.2)	0.017
CA125 level at diagnosis >35 U/mL	140 (73.3)	187 (78.9)	0.175
FIGO stage			0.001
Early	161 (78.9)	170 (64.9)	
Advanced	43 (21.1)	92 (35.1)	
Primary staging/cytoreduction			0.018
RD <1 cm	184 (91.5)	200 (84.0)	
RD ≥1 cm	17 (8.5)	38 (16.0)	
Front-line chemotherapy			0.234
Pt-PTX	152 (79.2)	176 (74.3)	
Pt-CTX	40 (20.8)	61 (25.7)	
Response to chemotherapy			0.385
CR/PR	166 (84.7)	199 (81.6)	
SD/PD	30 (15.3)	45 (18.4)	
Cycles of front-line chemotherapy			0.513
<6	56 (28.1)	64 (25.4)	
≥6	143 (71.9)	188 (74.6)	
PFI after primary chemotherapy			0.640
≥7 months	153 (73.2)	191 (71.3)	
<7 months	56 (26.8)	77 (28.7)	
Surgical resection after the first relapse			0.492
No	77 (77.8)	93 (73.8)	
Yes	22 (22.2)	33 (26.2)	
Chemotherapy after the first relapse			0.616
Pt-PTX	17 (37.8)	27 (33.3)	
Other	28 (62.2)	54 (66.7)	

CR, complete response; CTX, cyclophosphamide; FIGO, the International Federation of Gynecology and Obstetrics; OCCC, ovarian clear cell carcinoma; PD, Progressive disease; PFI, progression-free interval; PR, partial response; Pt, platinum; PTX, paclitaxel; RD, residual disease; SD, stable disease.

Differences in the continuous variables were tested using the Mann–Whitney *U* test.

Correlations between categorical variables were compared using Pearson's chi-square or Fisher's exact tests.

Table S2. Clinico-pathological characteristics of multi-institutional ovarian clear cell carcinoma (OCCC) patients with early- and advanced-stage disease.

Variables	Early stage n = 371	Advanced stage n = 150	P
Age >50 years	174 (46.9)	78 (52.0)	0.332
Menopause	175 (47.2)	80 (53.3)	0.846
CA125 level at diagnosis >114.5 U/mL	112 (30.2)	107 (71.3)	<0.001
Endometriosis			0.001
Presence	161 (48.6)	43 (31.9)	
Absence	170 (51.4)	92 (68.1)	
Primary staging/cytoreduction			<0.001
RD <1 cm	331 (96.8)	91 (65.0)	
RD ≥1 cm	11 (3.2)	49 (35.0)	
Front-line chemotherapy			<0.001
Pt-PTX	223 (60.6)	124 (83.8)	
Pt-CTX	107 (29.1)	8 (5.4)	
Response to chemotherapy			<0.001
CR/PR	314 (84.6)	82 (54.7)	
SD/PD	29 (7.8)	57 (38.0)	
Cycles of front-line chemotherapy			0.558
<6	90 (25.8)	40 (28.4)	
≥6	259 (74.2)	101 (71.6)	
PFI after primary chemotherapy			<0.001
≥7 months	307 (83.0)	64 (43.8)	
<7 months	63 (17.0)	82 (56.2)	
Surgical resection after the first relapse			<0.001
No	38 (48.1)	69 (74.2)	
Yes	41 (51.9)	24 (25.8)	
Chemotherapy after the first relapse			<0.001
Pt-PTX	39 (58.2)	15 (20.5)	
Other	28 (41.8)	58 (79.5)	

CR, complete response; CTX, cyclophosphamide; FIGO, the International Federation of Gynecology and Obstetrics; OCCC, ovarian clear cell carcinoma; PD, Progressive disease; PFI, progression-free interval; PR, partial response; Pt, platinum; PTX, paclitaxel; RD, residual disease; SD, stable disease.

Differences in the continuous variables were tested using the Mann–Whitney *U* test.

Correlations between categorical variables were compared using Pearson's chi-square or Fisher's exact tests.

Table S3. Univariate analysis of prognostic factors in the complete ovarian clear cell carcinoma (OCCC) cohort. (N = 536).

Variables		N	Progression events	Median time to progression (months)	HR for progression (95% CI)	Death events	Median time to death (months)	HR for death (95% CI)
Age	<50 years	274	95	166.9	1.00	61	NR	1.00
	≥50 years	258	88	NR	0.99 (0.74–1.32)	77	NR	1.37 (0.98–1.91)
CA125 level at diagnosis	< 114.5 U/mL	248	55	NR	1.00	33	NR	1.00
	≥ 114.5 U/mL	224	107	38.5	2.96 (2.13–4.11)	90	105.9	3.85 (2.58–5.75)
Endometriosis present	No	268	99	166.9	1.00	74	NR	1.00
	Yes	209	61	NR	0.78 (0.57–1.08)	41	NR	0.70 (0.48–1.03)
FIGO stage	Early (I/II)	371	83	NR	1.00	50	NR	1.00
	Advanced (III/IV)	150	97	7.9	5.60 (4.14–7.58)	88	27.1	7.28 (5.12–10.35)
Primary staging/ cytoreduction	RD <1 cm	429	131	NR	1.00	95	NR	1.00
	RD ≥1 cm	61	36	7.7	4.05 (2.76–5.92)	35	24.1	4.36 (2.95–6.45)
Front-line chemotherapy	Pt-CTX	120	34	NR	1.00	19	NR	1.00
	Pt-PTX	354	129	166.9	1.53 (1.04–2.24)	98	NR	2.04 (1.24–3.33)
Cycles of chemotherapy	<6	134	46	NR	1.00	44	NR	1.00
	≥6	369	126	166.9	0.89 (0.54–1.25)	85	NR	0.60 (0.41–0.86)
Response to chemotherapy	CR/PR	406	106	NR	1.00	66	NR	1.00
	SD/PD	86	59	2.9	9.11 (6.41–12.96)	62	14.1	11.37 (7.88–16.41)
PFI after primary chemotherapy	≥7 months	386	–	–	–	48	NR	1.00
	<7 months	150	–	–	–	92	16.2	18.27 (12.56–26.59)
Tumor resection after the first relapse	No	190	–	–	–	77	109.2	1.00
	Yes	67	–	–	–	32	70.3	1.12 (0.74–1.69)
Chemotherapy after the first relapse	Pt-PTX	55	–	–	–	25	120.3	1.00
	Other	89	–	–	–	58	25.6	2.45 (1.49–4.02)

CI, confidence interval; CR, complete response; CTX, cyclophosphamide; FIGO, the International Federation of Gynecology and Obstetrics; HR, hazard ratio; NR, not reached; OCCC, ovarian clear cell carcinoma; PD, Progressive disease; PFI, progression-free interval; PR, partial response; Pt, platinum; PTX, paclitaxel; RD, residual disease; SD, stable disease; y, years.

HRs and 95% CIs were estimated using Cox proportional hazards models.
